# Predicted Epitope Abundance Supports Vaccine-Induced Cytotoxic Protection Against SARS-CoV-2 Variants of Concern

**DOI:** 10.3389/fimmu.2021.732693

**Published:** 2021-11-25

**Authors:** Antonio J. Martín-Galiano, Francisco Díez-Fuertes, Michael J. McConnell, Daniel López

**Affiliations:** ^1^ Intrahospital Infection Laboratory, National Center of Microbiology, Instituto de Salud Carlos III, Majadahonda, Spain; ^2^ Acquired Immune Deficiency Syndrome (AIDS) Immunopathology Unit, Centro Nacional de Microbiología, Instituto de Salud Carlos III, Majadahonda, Spain; ^3^ Presentation and Immune Regulation Unit, Centro Nacional de Microbiología, Instituto de Salud Carlos III, Majadahonda, Spain

**Keywords:** SARS-CoV-2, escape mutant, HLA, vaccine, T cell epitope, epidemic

## Abstract

The effect of emerging SARS-CoV-2 variants on vaccine efficacy is of critical importance. In this study, the potential impact of mutations that facilitate escape from the cytotoxic cellular immune response in these new virus variants for the 551 most abundant HLA class I alleles was analyzed. Computational prediction showed that most of these alleles, that cover >90% of the population, contain enough epitopes without escape mutations in the principal SARS-CoV-2 variants. These data suggest that the cytotoxic cellular immune protection elicited by vaccination is not greatly affected by emerging SARS-CoV-2 variants.

## Introduction

Vaccine performance against current and future emerging SARS-CoV-2 strains is a challenging issue in the control of COVID-19 (1). Although vaccine prophylaxis is a critical factor in social protection and economic recovery against the pandemic, current formulations are based on the original D614 spike protein sequence of the Wuhan-1 wild-type strain. However, novel SARS-CoV-2 variants, and further sub-variants, are being increasingly detected (2). Changes in the amino-acid sequence of the spike protein present in these viruses, may affect several stages of the replicative cycle of the virus and/or efficacy of the immune response. It has been shown that humoral protection is retained by distinct vaccines against emerging viral lineages (3) (4) (5). However, vigorous activation of the three arms of adaptive immunity: neutralizing antibodies, virus-specific CD4^+^ T cells and IFN-γ-producing CD8^+^ T cells were identified after both SARS-CoV-2 natural infection and vaccination (6). Changes in the S protein between new SARS-CoV-2 variants compared to antigens included in current vaccines in use can lead to the selection of escape mutants.

In contrast to the flexibility of the epitope-HLA class I interaction, recognition of mutated peptides by the T cell receptor is rigid, where a single change in the epitope sequence can cause a total loss of antigen recognition. This extremely low tolerance to amino acid changes with respect to the sequence used for vaccination favors epitope escape at the T lymphocyte activation level, rendering previously activated lymphocytes useless. In support of this concept, confirmed cases of re-infection associated with different viral genotypes were detected at the beginning of the pandemic (7), and re-infection of immunized individuals by mutational evasion is common in other viruses such as influenza (8).

Altogether, the analysis of the influence of mismatches between the immune response elicited by existing vaccines and emerging variants is of primary importance. In this study, we have approached this aspect focused on the cytotoxic branch of the immune response. Although we observed some supertype-dependent differences in the predicted quality of cytotoxic protection against strains of concern, this effect is still minor compared to the global cytotoxic response elicited of current administrated vaccines.

## Methods

### SARS-CoV-2 Variants

All SARS-CoV-2 variants with at least 100 genomes for each month from January to May 2021 were retrieved from the GISAID’s emerging variant monitoring tool (**Supplemental Table S1**). These variants could become relevant due to signs of increased spread combined with potential effects on receptor or antibody binding. According to GISAID, there are currently 147 amino acid changes and deletions in the Spike protein that occur in at least 10 different geographical locations. These changes were identified in studies to cause antibody escape, increase ACE2 binding or increase Spike protein expression and stability. Thus, they are considered as part of mutation combinations or constellations forming potential variants to be monitored.

### HLA Class I Epitope Prediction and Analysis

Non-redundant HLA class I epitopes between 8-12 residues in the spike protein of the SARS-CoV-2 reference proteome (Wuhan-1; RefSeq: NC_045512.2) were predicted using NetMHCIpan EL 4.1 (9). Predictions were restricted to alleles that share anchor residues (labeled green and white in the original publication) of the 551 alleles including in the twelve HLA class I supertypes (10). Binding epitopes were considered those that satisfy the rank ≤ 0.5 and score ≥ 0.5. For redundant epitopes, those sharing the same binding core for the same allele, only the one with the highest score was considered per allele. Non-redundant epitopes were further verified through the NetMHCIpan BA 4.1 algorithm (9), applying an IC50 ≤ 500nM threshold. These verified non-redundant epitopes did not match any of those predicted for a random sequence with the same length and residue composition than the reference SARS-CoV-2 spike protein generated with the EXPASY RandSeq tool (https://web.expasy.org/randseq/). To further test the specificity and sensitivity of NetMHCIpan EL 4.1 algorithm, substitution of all Pro and Arg/Gln by Ala yielded no epitopes for HLA-B*07:02 and HLA-B*27:05 alleles, respectively, as these amino acids are their respective anchor motif residues. Progressive random position datasets were produced by selection of numbers between 1 and 1273, the spike protein residue length, by the perl rand function. Iterative selection was carried out on the remaining non-selected positions after completing the 1273 positions. The average escape mutation rate for each supertype is the percentage of mutated epitopes for all alleles in the supertype considering all the mutations in each (sub)variant.

## Result and Discussion

While the study of escape mutants that affect the antibody response can be carried out experimentally, the global cellular immune response consisting of CD4^+^ T lymphocytes and especially CD8^+^ T cells can only be approached bioinformatically due to the large number of HLA class II, and especially, HLA class I alleles distributed in the human population. However, many HLA class I molecules identified to date have been grouped first in families, and later in twelve canonical types sharing strong similarities at the peptide-ligand specificity level, termed supertypes, that cover >90% of the world population regardless ethnicity (10). These accounted for 551 alleles that experimentally shared the same motifs within the supertype plus those alleles showing exact matches for residues at the specific B and F pockets of the HLA molecule. While more than 17,000 HLA class I alleles have been reported (11), the utilization of supertypes dramatically reduces data complexity and facilitates assessment of herd immunity. Thus, first we computationally predicted the theoretical epitopes from the SARS-CoV2 spike protein, the only protein included in internationally licensed vaccines, that could be presented by each of the 551 supertype-associated HLA class I alleles.

The latest version (v4.1) of the universal and neural network-based netMHCpan EL algorithm was utilized, which outperforms any other method so far (9) and is thus the one recommended by the central Immune Epitope Database and Analysis Resource (12). To prevent the prediction of large epitope datasets that are enriched in weak and non-binders, the same or more stringent thresholds that those considered in recent SARS-CoV-2 studies that used the same algorithm [see for instance (13, 14)] were applied. While netMHCpan series does not explicitly consider antigen proteolytic processing by the (immuno)proteasome or suitability as TAP transporter substrates, the EL version of the algorithm has been trained with real peptide binders, which largely palliates these limitations (9). In addition, the epitope pool was re-analyzed through the BA version of the algorithm, which has been trained with a fully unrelated dataset and does not provide arbitrary units but a biochemical-meaning estimation of the binding. It was found that 82.8% of the initially EL predicted epitopes showed IC50 ≤ 500nM to their respective alleles, associated with strong-to-intermediate affinity. This slightly reduced set of consensus supertype epitopes were taken for assays hereafter (**Supplemental Table S2**).

**Figure 1:**
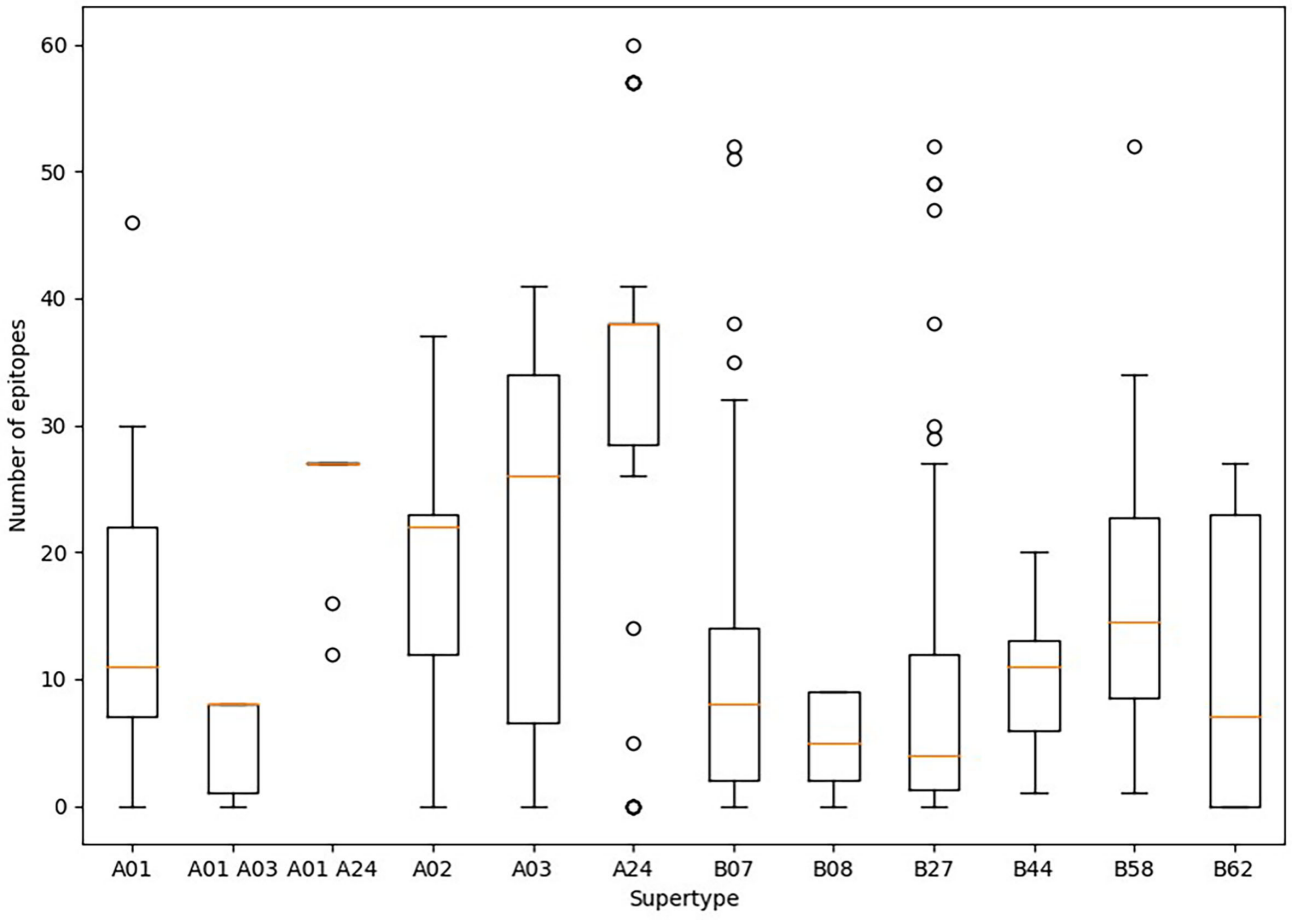
Average number of epitopes in the Wuhan-1 spike protein sequence predicted for HLA class I alleles including in the 12 supertypes. Orange dash indicated the median value. Box limits indicate the interquartile range. Whiskers are adjusted to maximal and minimal values if lower than 1.5 times the IQR. Further outliers are indicated as circles.

Predicted ligands for most HLA class I supertype molecules reached a few tens per allele but in two cases (**Figure 1**). First, a majority of HLA class I alleles from the A24 supertype could bind more than 30 epitopes (**Figure 1**). In contrast, the number of predicted epitopes for most HLA class I molecules included in both “A01 A03” and B08 supertypes was very low, *i.e.*, less than 10 epitopes per allele (**Figure 1**). These “A01 A03” and B08 supertypes, besides some individual alleles of other supertypes that also showed low predicted epitope binding, (**Figure 1**) would be candidates to be affected by escape mutants within SARS-CoV-2 variants.

**Figure 2:**
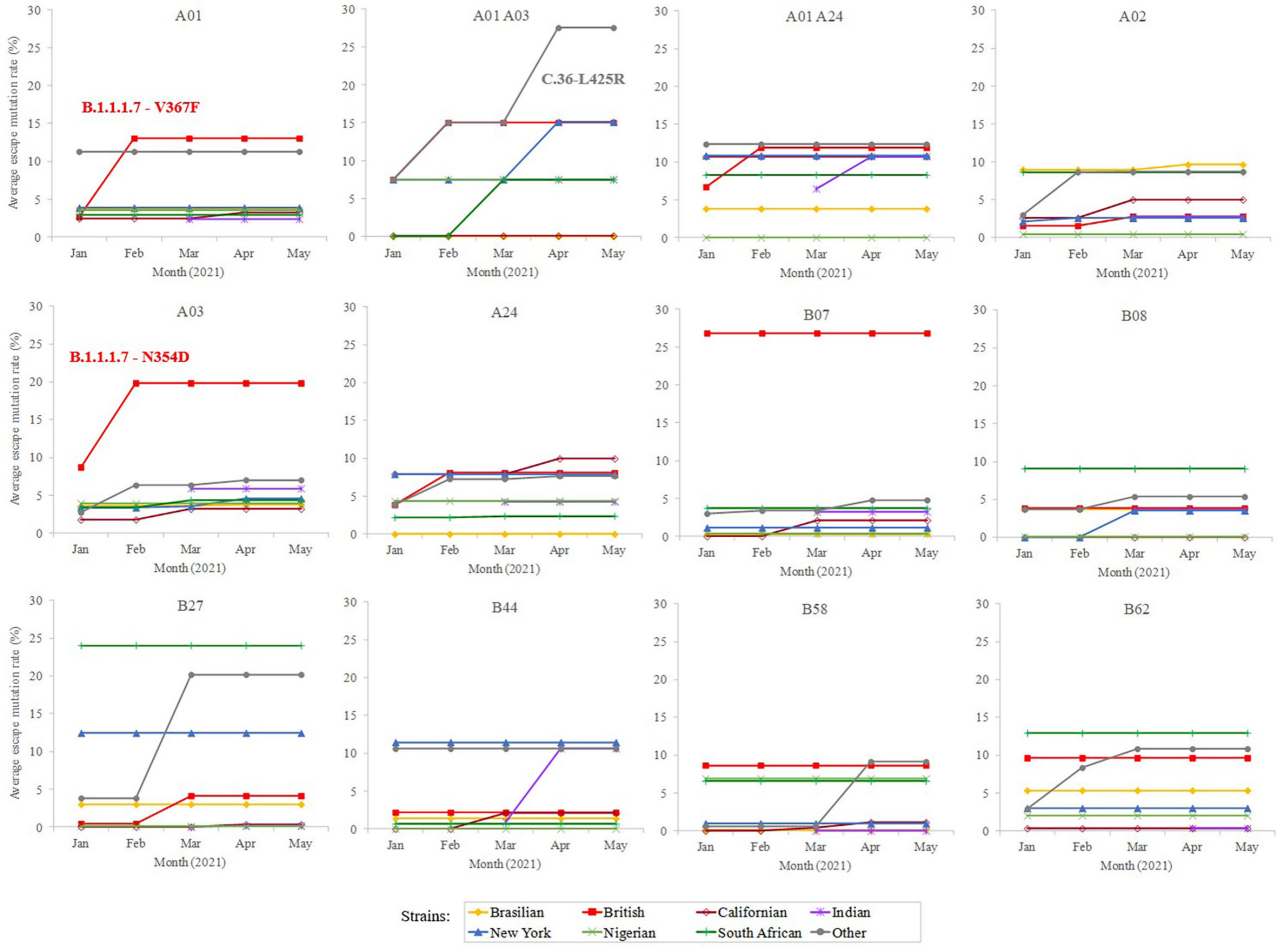
Average escape mutation rate by the sub-variant of the SARS-CoV-2 variants of concern. Only the sub-variant with the highest value is indicated. Sub-variant and emerging mutation is indicated if produce an increment of >3% rate within the strain and achieve the top value.

In addition, a predictive analysis of the impact of non-synonymous mutations described in the bibliography in 2020 over the supertype HLA class I alleles was carried out. Most of the SARS-CoV-2 variants described in the past year underwent little loss of epitopes in the protein spike. In these strains, the average escape epitope rate was lower than 10%, except for some British and South Africa variants, which affected the B07 and B27 supertypes, respectively (**Figure 2**).

To study whether potential mutations have arisen from the beginning of massive vaccination, a total of 364 unique emerging variants defined by GISAID (15) (https://www.gisaid.org/) during January-May, 2021 were considered. This isolate dataset covered the variants of concern and of interest, as defined by the WHO, besides their respective sublineages. The number of spike residue changes in new SARS-CoV-2 variants was still low (≤ 8 mutated residues) (**Supplemental Table S1**), with a moderate increase (<25% escape epitopes except for in two cases) in the average rate of epitope escape mutation per allele and supertype (**Figure 2**).

Among the strains of concern, the B.1.1.7-V367F variant increases the average epitope escape rate for the A01 supertype from 2.7 to 13.2% due to the V367F mutation, also detected in the A.23.1 variant. The C.36-L452R variant leverages the “A01 A03” epitope escape rate by the R346S change from 15.0% to 27.5%. The B.1.1.7-L354D sub-variant increased for A03 escape rate from 8.9% to 19.9%. Finally, the B.1.1.7-N354D sub-variant increased A03 escape rate from 8.7% to 19.8% through the N354D change. More specifically, the eight predicted SARS-CoV-2 S protein epitopes presented by HLA-A*30:01 from the “A01 A03” supertype generated by immunization with current vaccines would be reduced to just six epitopes if the SARS-CoV-2 C.36-L452R variant were the infectious agent in individuals carrying this HLA class I molecule (**Supplemental Table S2**). HLA-A*30:01 allele frequency is greater than 10% in diverse populations of Guinea Bissau, Mali, Zambia, Israel, Pakistan, and in the predominant Han ethnic group of China. Remarkably, the only epitope predicted by SARS-CoV-2 vaccines associated with HLA-A*30:08 was mutated in this C.36-L452R variant generating an absolute invalidation of this allele in the cytotoxic immune response memory induced by vaccination (**Supplemental Table S2**). In addition, all supertypes included several HLA class I molecules with very few epitopes predicted against the SARS-CoV-2 spike protein (**Figure 1**). These 83 HLA class molecules included in different supertypes with less than 2 predicted epitopes derived from SARS-CoV-2 vaccines showed significant loss (even up to the total) of epitopes when new virus variants were analyzed (**Supplemental Table S2**). In all of these HLA alleles severely diminished or even total absence of the protective cytotoxic T cell response associated with the vaccine would be expected. For example, HLA-B*35 do not bind HIV peptides and fail to mediate a protective response in HIV-infected individuals involving rapid AIDS progression among individuals with this allele (16).

**Figure 3:**
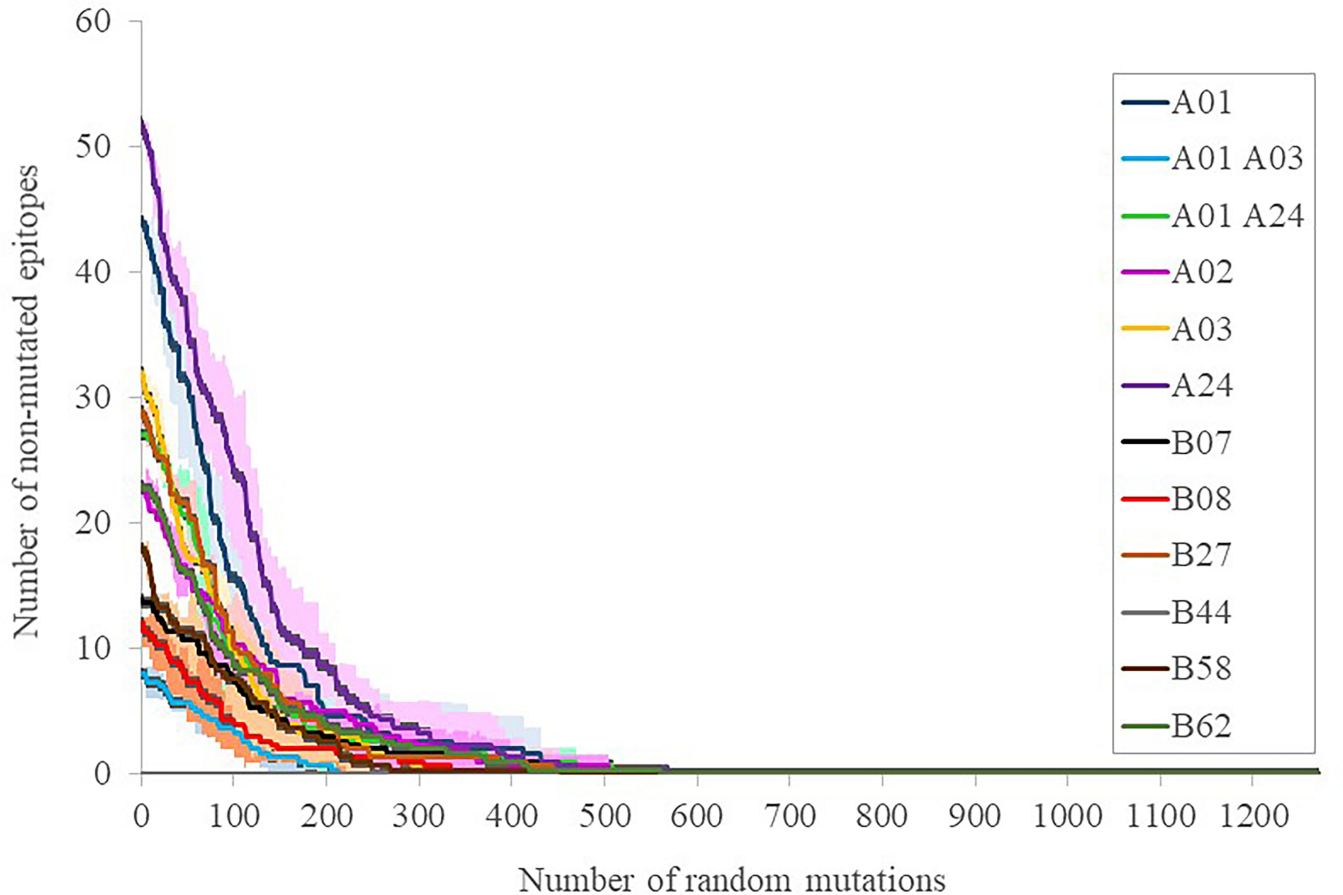
Number of intact epitopes after increasing random mutagenesis. The number of intact epitopes for representative alleles of the twelve supertypes was calculated after random progressive position sets ranging from 1 and 1273 positions, i.e. the length of the full spike protein (**Supplemental Table S3**). Representative supertype alleles were selected by prioritizing the first numeric allele one of the same family of the supertype name included in (10): A*01:01 (A01), A*30:01 (A01 A03), A*29:02 (A01 A24), A*02:01 (A02), A*03:01 (A03), A*24:02 (A24), B*07:02 (B07), B*08:01 (B08), B*27:02 (B27), B*44:02 (B44), B*58:01 (B58) and B*15:01 (B62). The averages (lines) of three simulations ± SD (shades) for the epitopes of each supertype representative allele are shown.

To calibrate the general influence of the number of mutations on the maintenance of the elicited response, three sets of progressive random mutations were generated and the alteration of intact epitopes assessed (**Figure 3**). This assay is based on the worst possible scenario assumption that any substitution in any epitope, while it does not necessarily negate binding to the MHC I molecule in the presenting cell, does perturb the exquisite TCR recognition by memory CD8+ lymphocytes (17). By doing that, 39 ± 11, 81 ± 10 and 141 ± 20 mutations (average ± SD, for 12 supertype representative alleles) caused that only 75%, 50% and 25% of the original epitopes, respectively, remained (**Figure 3**). These mutational levels are much higher than those found in any strain of concern, but still far smaller than those from the closest SARS-CoV-1 (304 mutated residues, reference Tor2 strain, NCBI id: NC_004718.3, Protein: P59594.1) and MERS-CoV (870 mutated residues, reference HCoV-EMC/2012 strain, NCBI id: NC_019843, Protein: YP_007188579.1) clinical entities. We therefore estimate that, even when mutations with respect to SARS-CoV-2 in these species are not evenly distributed, the escape of the cytotoxic response elicited by current vaccines in most humans requires mutational ciphers that fall halfway between current strains and SARS-CoV-1. Variant selection in this respect may incur fitness incompatibility unless a large evolutionary leap takes place, an extreme that has not been observed at least in the known genomic space of the virus. Notably, T-cell cross-responses have been consistently suggested even for seasonal common cold coronaviruses, however, it rather involves the less strict MHC II-driven CD4+ lymphocytes (18).

A central issue is to what extend these mutations may be selectively favored in potentially emerging cytotoxic escape sub-strains. For that, mutation rates in epitopic and non-epitopic random nonamers were compared in the most emerging-escape substrain for each supertype and strain combination. As negative controls, between 61 and 131 non-epitopic non-redundant background nonamers, i.e. negative control peptides were identified for each supertype. Notably, no mutation occurred in control background peptides for eleven strain-supertype combinations (**Table 1**). Two prominent variants in this respect were the B.1 variant characterized by Δ242-244, K417N, N501Y, D614G, and A701V, showing 8.7%, 20.2% and 9.7% epitope escape rates for A02, B27 and B62 supertypes, respectively; and the B.1.1.7-N354D variant showed a 19.8% epitope escape rate for the A03 supertype. In addition, proportional increases of over 5-fold between epitopic and non-epitopic peptide mutation rates were detected for at least one variant in eleven out of the twelve supertypes. Four of these variants showed epitope escape rates over 20% for “A01 A03”, B07 or B27 supertypes (**Table 1**).

**Table 1:** SARS-CoV-2 variants showing enrichment epitope mutation rate.

Supertype	SARS-CoV-2			% epitope	% control peptide	Epitope/control
	Top Lineage	Variant^a^	Top Location	mutation rate	mutation rate	mutation rate ratio
A01	B.1.1.7	69X_70X_144X_367F_501Y	45 Scotland	13.02	1.64	7.94
A01	A.23.1	367F_681R	112 Uganda	11.27	1.64	6.87
A01 A03	C.36	69X_70X_346S_452R	61 England	27.50	0.78	35.26
A01 A03	B.1.1.7	69X_70X_144X_384L_501Y	108 England	15.00	1.56	9.62
A01 A03	B.1.525	5F_144X_484K	73 New York	15.00	1.56	9.62
A01 A24	B.1.427	452R_503I	51 Minnesota	14.53	0.00	–
A01 A24	A.23.1	367F_681R	112 Uganda	12.35	0.90	13.72
A01 A24	B.1.525	144X_452R	152 New York	10.73	0.90	11.92
A01 A24	B.1.1.7	69X_70X_144X_367F_501Y	45 Scotland	11.88	1.80	6.60
A01 A24	B.1.617.2	452R_478K_681R	1029 England	10.73	1.80	5.96
A02	B.1	242X_243X_244X_417N_501Y	97 Turkey	8.68	0.00	–
A02	P.1	18F_417T	19 Massachusetts	9.62	1.45	6.63
A03	B.1.1.7	69X_70X_144X_354D_501Y	109 England	19.83	0.00	–
A24	B.1.525	144X_452R	152 New York	7.92	0.00	–
A24	A	449H_501Y	51 England	7.67	0.00	–
A24	B.1.427	452R_522P	195 Michigan	9.95	1.18	8.43
B07	B.1.1.7	69X_70X_144X_501Y	300 England	26.81	1.39	19.29
B27	B.1	242X_243X_244X_417N_501Y	97 Turkey	20.17	0.00	–
B27	B.1.525	144X_452R	152 New York	12.44	1.54	8.08
B27	B.1.351	18F_242X_243X_244X_417N_484K_501Y	255 Mamoudzou	24.04	3.08	7.81
B44	A.23.1	367F_681R	112 Uganda	10.61	0.00	–
B58	C.36	69X_70X_346S_452R	61 England	9.15	1.19	7.69
B62	B.1	242X_243X_244X_417N_501Y	97 Turkey	9.70	0.00	–
B62	B.1.1.7	69X_70X_144X_501Y	300 England	13.86	2.00	6.93
B62	B.1.351	18F_242X_243X_244X_417N_484K_501Y	255 Mamoudzou	11.88	2.00	5.94

a Variants carrying >5% epitope mutation rate for at least one supertype, besides no mutated control epitope or >5-fold enrichment respect to background peptides are shown. Only the top variant per lineage is selected.

Given that no more than eight residue changes have been observed in the spike proteins of clinically relevant variants, it appears unlikely that these mutations *per se* will achieve critical mass in the short term to be subjected to selective pressure by cytotoxic evasion for most alleles. Nevertheless, they may be associated with selection by other factors such as transmissibility, as suggested by epidemiological data for the principal strains of concern (19–21). In this context, N354D and V367F increase affinity for the hACE2 receptor, as assessed by molecular dynamics (22), whereas R346S provides escape from some monoclonal antibodies (23). The presence of these mutations may therefore add a secondary partial effect that is co-selected by other factors.

**Figure 4:**
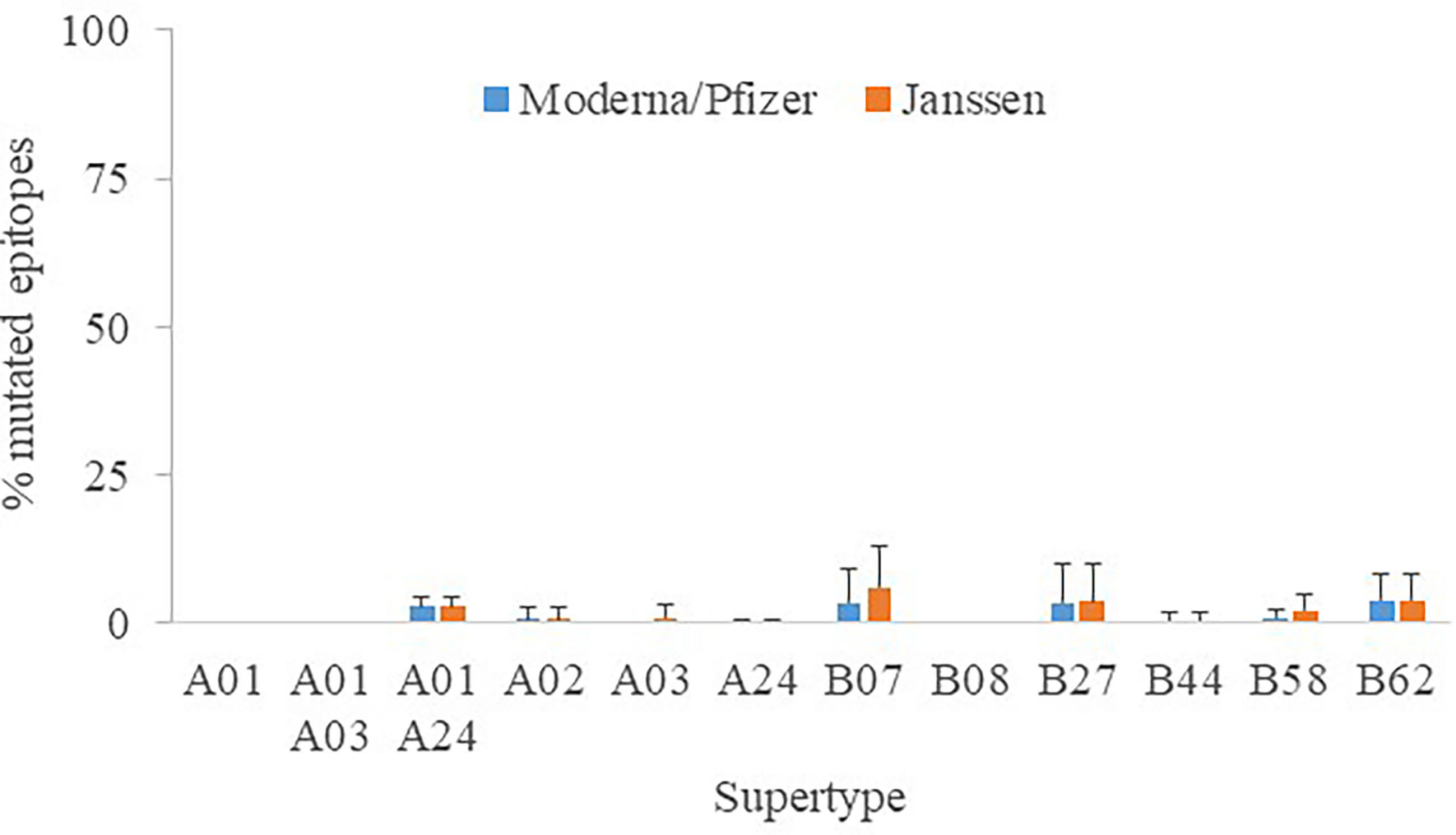
Percentage of mutated epitopes by vaccine antigen modifications. The average percentage of mutated epitopes from all alleles in a supertype, plus standard deviation, is shown.

Another focal point is whether distinct vaccine formulations may have an effect on peptide recognition. For the sake of universality, all assays were conducted with the original Wuhan-1 spike sequence, which have been unmodified by Astra Zeneca AZD1222, Sputnik V and SinoVac (CoronaVac) antigens. In contrast, K986P/V987P mutations were engineered to stabilize the close conformation and enhance the humoral protection (24) in Moderna mRNA-1273, Pfizer BNT162b2 and Janssen Ad26.COV2.S antigens, the latter further contained two changes involving the arginines of the furin site to diminish proteolysis (25). The epitopes destroyed by these manipulations reached 0 - 3.9% (supertype averages) for Moderna/Pfizer and 0 - 6.1% for Janssen protein versions (**Figure 4**). This mostly affected alleles of the B07 supertype, but never involving more than four epitopes. Hence, the influence of the vaccine antigen may be deemed minor and the results stated here essentially taken as standard for all vaccines.

In conclusion, HLA class I molecules of most common human alleles can present enough unmodified ligands from the principal SARS-CoV-2 variants to activate vaccine-generated CD8+ lymphocytes, even when the strains of concern analyzed represent a tiny fraction of the whole genomic space of the virus, since these strains of concern are circulating explosively throughout the world. This may subsequently lead to the efficient elimination of the infected cells. We envisage the number of required mutations to escape vaccine-driven CD8^+^ activation is so high that it is most likely antagonic to spike protein function. During the revision course of our study, other authors confirmed a very moderate reduction, if any, of the memory CD8^+^ response for ligands of the principal strains of concern in a limited number of vaccinated subjects (26). However, it is still uncertain how key mutations associated with other selection factors in some emerging sub-variants may be relevant for individuals with different HLA alleles, mainly those included in some supertypes, such as “A01 A03” and B08. In particular, it may be dramatic for those patients unable to mount a protective humoral response upon vaccination or impoverished T lymphocyte pools. As immunoprotection is provided *via* activation of the humoral, helper and cytotoxic pathways, impairment of one or more of these branches by viral mutations may lead to vaccine failure. It is conceivable that new emerging highly-transmissible strains carrying novel mutation pools arise in future. Several initiatives aim to integrate diverse information sources to monitor SARS-CoV-2 epidemiology in real time (27). Given the high importance of the CD8^+^ T lymphocytes in the memory response, we propose that mutations in HLA class I ligands negating CD8+ lymphocyte activation in vaccinated individuals should be tightly surveyed for alleles with scarce SARS-CoV-2 epitopes.

## Data Availability Statement

The original contributions presented in the study are included in the article/**Supplementary Material**. Further inquiries can be directed to the corresponding authors.

## Author Contributions

Conception: DL. Experimental design: AM-G and DL. Acquisition of data: AM-G and FD-F. Analysis of data AM-G, MM, and DL. Manuscript preparation: AM-G and DL. Manuscript revision: AM-G, FD-F, MM, and DL. All authors contributed to the article and approved the submitted version.

## Funding

This research was supported by grants from COV20_00679 (MPY 222-20), to MM, MPY 509/19 to AM-G, and MPY 388/18 to DL of “Acción Estratégica en Salud” from the ISCIII.

## Conflict of Interest

The authors declare that the research was conducted in the absence of any commercial or financial relationships that could be construed as a potential conflict of interest.

## Publisher’s Note

All claims expressed in this article are solely those of the authors and do not necessarily represent those of their affiliated organizations, or those of the publisher, the editors and the reviewers. Any product that may be evaluated in this article, or claim that may be made by its manufacturer, is not guaranteed or endorsed by the publisher.
